# Physical processes controlling the rifting of Larsen C Ice Shelf, Antarctica, prior to the calving of iceberg A68

**DOI:** 10.1073/pnas.2105080118

**Published:** 2021-09-27

**Authors:** E. Larour, E. Rignot, M. Poinelli, B. Scheuchl

**Affiliations:** ^a^Jet Propulsion Laboratory, California Institute of Technology, Pasadena, CA 91109;; ^b^Department of Earth System Science, University of California, Irvine, CA 92697;; ^c^Department of Geoscience and Remote Sensing, Delft University of Technology, 2628 CN, Delft, The Netherlands

**Keywords:** Larsen C, Antarctica, sea level, ice shelf, fracture

## Abstract

The sudden propagation of a major preexisting rift (full-thickness crack) in late 2016 on the Larsen C Ice Shelf, Antarctica led to the calving of tabular iceberg A68 in July 2017, one of the largest icebergs on record, posing a threat for the stability of the remaining ice shelf. As with other ice shelves, the physical processes that led to the activation of the A68 rift and controlled its propagation have not been elucidated. Here, we model the response of the ice shelf stress balance to ice shelf thinning and thinning of the ice mélange encased in and around preexisting rifts. We find that ice shelf thinning does not reactivate the rifts, but heals them. In contrast, thinning of the mélange controls the opening rate of the rift, with an above-linear dependence on thinning. The simulations indicate that thinning of the ice mélange by 10 to 20 m is sufficient to reactivate the rifts and trigger a major calving event, thereby establishing a link between climate forcing and ice shelf retreat that has not been included in ice sheet models. Rift activation could initiate ice shelf retreat decades prior to hydrofracture caused by water ponding at the ice shelf surface.

## Significance

The stability of Antarctica and its contribution to sea-level rise are determined by the evolution of its ice shelves, which are vast expanses of floating ice that buttress the continent. Ice shelves have been undergoing major changes in recent decades, many of them collapsing. The presumption is that these events are caused by hydrofracturing and unusual wave forcing. We find that a main control on fracturing is the thickness of the ice mélange encased in and around preexisting rifts that penetrate the entire ice shelf thickness. If the ice mélange thins beyond a threshold value, the rifts reactivate and trigger iceberg calving. This process linking climate forcing and ice shelf retreat is missing from models and does not require hydrofracture.

The Larsen A and Larsen B ice shelves, in the Antarctic Peninsula, collapsed in spectacular fashion in 1995 and 2002, respectively, as a result of climate warming (1, 2). While the loss of the Larsen A and B ice shelves did not impact sea level directly, it affected their upstream glaciers in a major way (3). The Larsen A and Larsen B glaciers experienced a three- to eightfold acceleration in speed following the collapse of these buttressing ice shelves (4–7), which increased land ice discharge into the ocean and contributed to sea level rise from the Antarctic Peninsula. These two events demonstrated the importance of ice shelf buttressing and exemplified what could happen elsewhere in Antarctica as climate warming extends farther south. If all Antarctic glaciers with ice shelves were to accelerate eightfold, sea level would rise 4 m per century.

**Fig. 1.**
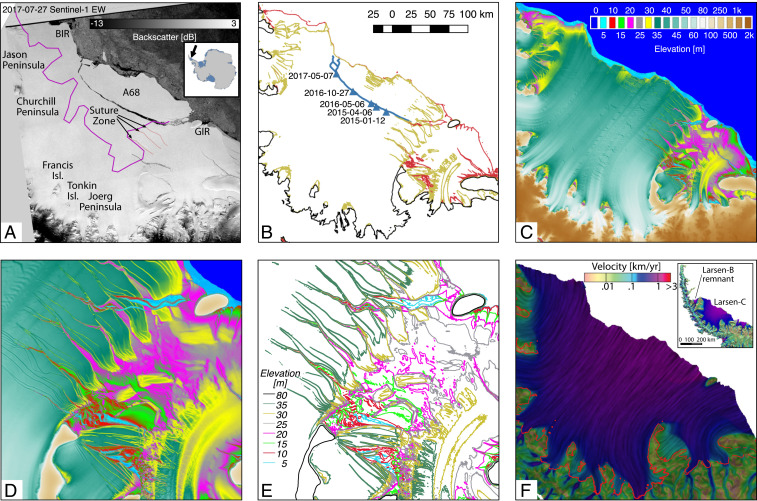
(*A–F*) Larsen Ice Shelf, Antarctic Peninsula with (*A*) backscatter image from Sentinel-1 EW on 27 July 2017 (62) with *Inset* map for location in Antarctica and the location of the compressive arch of stability of the ice shelf (purple); (*B*) color-coded 20- (red), 40- (brown), and 100-m (black) contour levels in surface elevation with time-tagged (blue triangles) position of the rupture tip of A68 from Sentinel-1a interferometric SAR; (*C*) surface elevation above mean sea level (in meters) from TanDEM-X digital elevation model in years 2013 and 2014; (*D*) zoom-in around GIR; (*E*) contour levels color coded from 5 to 80 m in 5-m increments; and (*F*) surface speed (in meters per year) on a logarithmic scale color coded from slow (brown) to fast (blue/red) (63) with grounding line position (red) from ERS-1/2 differential SAR interferometry (64).

The Larsen C Ice Shelf, immediately south of Larsen A and B (Fig. 1), is the largest ice shelf in the Antarctic Peninsula (46,465 ${km}^{2}$). It drains a land area of 18,120 ${km}^{2}$, with an ice flux of 14.5 Gt/y and an ice volume equivalent to a global sea level rise of 0.9 cm (8). As warming continues, Larsen C is expected to collapse (9). While Larsen C does not hold back a large volume of land ice, it stands north of the Ronne Ice Shelf, one of the largest ice shelves in Antarctica, which buttresses glaciers with a 158-cm sea level rise equivalent, or orders of magnitude larger than those in the Antarctic Peninsula. Addressing the fate of the Larsen ice shelves is therefore an issue of considerable importance for sea level rise from Antarctica.

The prevailing view for explaining the evolution of Larsen A and B and their collapse is the hydrofracture theory (10–13). In this theory, melt water accumulates at the surface of an ice shelf with sufficient warming, collects in cracks, and refreezes at depth at the end of the melt season, which results in further cracking of the ice shelf. Melting ponds have been observed in the Eastern Antarctic Peninsula during warm summers throughout the 20th century (14–16). Using Landsat and Earth Remote Sensing (ERS)-1/2, melt ponds were identified in the northern section of Larsen B and on Larsen A in the summer of 1988 and more evidently in 1993 (10, 17), 2 y before the collapse in January 1995 (1). In the late 1990s, warmer summers and enhanced melting seasons (18, 19) spread meltwater ponds southward to reach their southernmost extension of 1999 just north of Cape Disappointment (11). As the melting season lengthened (20), melt ponds were observed through the entire Larsen B Ice Shelf until its collapse in March 2002 (21). The hydrofracture theory, however, does not explain why the ice front of Prince Gustav Channel Ice Shelf, north of Larsen A, started to retreat as early as 1957, Larsen A Ice Shelf started to retreat in 1975, and Larsen B Ice Shelf in 1986, i.e., decades before their collapse (16, 17). Similarly, the hydrofracture theory does not explain why A68 calved in the middle of the Antarctic winter, in the absence of melt water.

Calving events on ice shelves dominantly originate from ice front-parallel rifts that propagate in a direction transverse to the ice flow (22–25). When the ice blocks detach from the shelf, they form tabular icebergs. Iceberg production for Larsen C averages 31 Gt/y, or twice the grounding line flux. The ice shelf also loses mass from the bottom in contact with warm, salty ocean waters at a rate of 21 Gt/y (26). The initiation and propagation (or arrest) of rifts exert a major control on iceberg production and therefore on ice shelf mass balance (27).

Several studies have attempted to quantitatively couple rift growth (followed by ice breakup) to its destabilizing effect on Antarctic ice shelves, although calving processes are not well understood and modeled (see ref. 28 for a review on calving criteria). The “compressive arch” concept was introduced during an analysis of the strain rate distribution in the Larsen A Ice Shelf before its collapse in 1995 (23). In this theory, if the ice front breaks through a compressive arch, where only the least principal strain-rate component is compressive, the ice front retreat becomes irreversible. Fractures propagating seaward of the arch, where both principal strain-rate components are extensive, do not pose a risk to ice shelf stability. As pointed out elsewhere, ice shelf fractures tend to strike in a direction perpendicular to the ice flow and their propagation rates are maximized when the first principal stress and the fracture strike form a right angle (24). The distribution of angles between ice flow direction, principal stress component, and rift orientation can be used as an indicator of ice shelf stability (24, 29, 30).

Ice shelves are composed of meteoric units fed by inland glaciers, glued together along suture zones. Suture zones in Larsen C form seaward of the Joerg and Churchill Peninsula and around Tonkin and Francis Islands, in places where the ice shelf rifts apart from stress singularities along the coastline (Fig. 1). These fractured areas get filled with marine ice (31, 32), which accumulates in the downflow direction and progressively heals the fractures over time on time scales of decades to centuries. A similar infill accretes in between rift flanks, which are full-thickness cracks in the shelf (33). Depending on the exposure of ice fractures to the ocean and atmosphere freezing, suture zones and rifted areas are filled by a heterogeneous mixture of accreted ice, blown snow, and iceberg debris termed ice mélange. This ice mélange builds up over time into a thick, mechanically resistant and cohesive material (34–38). Areas filled by ice mélange are softer, warmer, and less prone to favor rift propagation than cold meteoric ice (9, 39–41). Rifts often stop propagating when they reach these suture zones (32, 42, 43).

Prior work on Larsen B and C has shown that melting of accreted ice in rifted areas may alter the longitudinal stress orientation on the ice shelf, facilitating rifting through suture zones and potentially destabilizing the ice shelf (24, 29, 32). As a consequence, the distribution of accreted ice (marine ice underneath shelves and ice mélange in atmosphere exposed areas), modulated by oceanic and atmospheric forcings, may be a link between climate forcing, rift propagation, and ice shelf retreat. Prior work also evaluated the impact of ice rigidity, ice fabric, and ice damage in controlling rift initiation or propagation (36, 44–48). Other studies have correlated the incidence of ocean swells or tsunamis to rifting episodes by using a combination of satellite monitoring and in situ seismometers (49–54). Ocean waves of sufficient energy and suitable period impact the ice front and induce cyclical flexural stresses to the ice shelves (25, 55–57). Excessive shelf bending in response to infragravity (period of ground swells) or longer period waves (storms or tsunamis) is speculated to cause fatigue damaging, hence controlling the onset of crack initiation and propagation (27, 51, 58). The effective impact of waves on ice shelves is substantially modulated by the presence of sea ice in the vicinity of the ice front, which forms a buffer layer that dissipates wave energy (59). Loss of this protective layer exposes ice shelves to the arrival of large swells or tsunamis, which may trigger calving and potentially cause ice shelf disintegration (60). Similar to marine ice and ice mélange in fractured areas, a warming climate curtails sea ice distribution, which exposes ice fronts to enhanced wave-induced stress.

When combined together, processes such as hydrofracture and viscoelastic flexure of ice shelves can lead to runaway processes such as iceberg-capsize tsunamigenesis as described in ref. 61, potentially leading to catastrophic collapse of ice shelves. Coupling between vertical bending of an ice shelf and horizontal stresses such as lateral shearing and longitudinal expansion is, however, currently poorly described. In particular, little is known about how the tsunamigenesis type collapse of an ice shelf competes or coexists with a scenario of increasing horizontal weakening of an ice shelf such as observed prior to the Larsen A collapse.

Iceberg A68 calved after the along-front propagation of a crack that had grown since 2005, stayed dormant for 10 y, opened up around 2014, stopped, and reopened up again in November 2016 (29, 30), to culminate with the release of A68 on 12 July 2017 (Fig. 1*B*). To put this event in context, the ice volume of A68 is equivalent to 42 y of calving history of Larsen C and brought its ice front into its farthest back position since the discovery of Larsen C by Captain Carl Arton Larsen in 1893. The ice front is now closer to the compressive arch of stability of the ice shelf than in the past century. How this event unfolded and how it relates to climate warming are essential elements to understand to model the evolution of Antarctic ice shelves in a warming climate.

The first phase of rift propagation in year 2014 stopped at the suture zone downstream of Joerg Peninsula (32, 43) that includes a significant fraction of ice mélange (Fig. 1 *C*–*E*). In the second phase, starting in November 2016, the rift opened up again and progressed rapidly across the ice front (Fig. 1*B*), growing mostly orthogonal to maximum tensile stresses (30). As of this date, no physical process has been proposed to explain the reactivation of the rift in November 2016. At the end of the calving, the ice front was in its most retreated position since first being discovered in 1893 and closest to the compressive arch (Fig. 1*A*) derived from ice velocity data collected from 2007 to 2009.

Here, we present a stress-balance analysis of the state of the ice shelf prior to the propagation that includes rifts. We evaluate the relationship between ice thinning and the opening rate of A68. We consider two major effects: 1) ice shelf thinning, which has been documented elsewhere (65), and 2) thinning of the ice mélange encased in the fractured sections of the ice shelf within and around the rifts, which has not been well studied. We use the modeling results to conclude on the impact of climate forcing on ice shelf rifting, ice shelf calving and retreat, and the future of Larsen C Ice Shelf and other Antarctic ice shelves.

## Model

We model the evolution of ice shelf stress balance around actively propagating rifts as the ice shelf and/or ice mélange thickness changes. Irrespective of the propagation criterion, i.e., linear elastic fracture mechanics (40, 41, 66, 67), damage mechanics (30, 47), or other physical representation of propagation processes, a metric for determining whether a rift propagates or not is the rate at which its flanks are moving away from each other (24, 44, 68), which we refer to here as the “opening rate.” The opening rate may be realistically represented as in ref. 69 via the modeling of active cracks/rifts as zero-width singularities into a shallow-shelf approximation (SSA) (70, 71) formulation of ice flow using the ice-sheet and sea-level system model (ISSM) (72).

Boundary conditions at the flanks of the rift depend on whether the rift is 1) actively opening, in which case water or mélange pressure applies as a normal boundary condition and we assume zero lateral shear, or 2) closing, in which case a nonpenetration normal boundary condition is applied [relying on application of contact mechanics (73) and penalty methods (74)] along with lateral shear (using a linear viscous friction law; see ref. 69 for details). The aim of this formulation is to solve for both the ice shelf flow velocity and the opening rate of each embedded rift. If we average the opening rate starting from the rupture tip along the main axis of each rift to the end of the rift, we obtain an average opening rate, which is an indicator of the propagation rate. This approach is similar to the Linear Elastic Fracture Mechanics (LEFM) when computing the stress intensity factor of a crack under displacement control instead of under stress control (75). Here, our hypothesis is that the rift propagates under displacement control because the stress field is similar across the rifts near Gibbs Ice Rise (GIR). Using this approach, we obtain a forward simulation of how changes in ice thickness, a major control on ice flow rates, impact rift opening rates and, in turn, control rift stability.

## Data and Model Setup

**Fig. 2.**
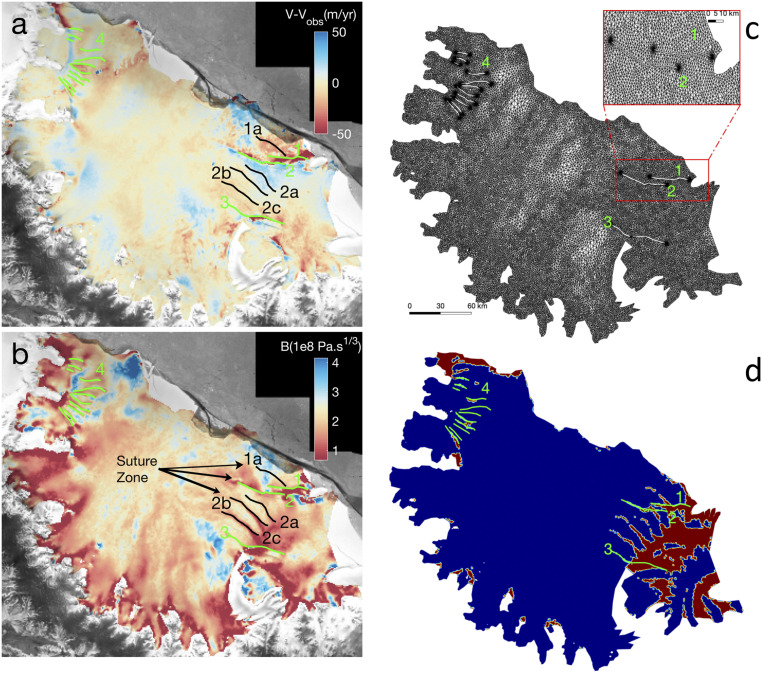
(*A* and *B*) Model results for Larsen Ice Shelf, Antarctica with (*A*) misfit between observed and modeled surface velocity (in meters per year) with active (green) and passive (black) rifts (full-thickness cracks) after inversion for (*B*) ice shelf rheology, B (in Pa⋅s^1/3^) using a control method, with the major rifts labeled 1 to 4 in green (and inactive rifts labeled 1a, 2a, 2b, and 2c in black). (*C* and *D*) The finite-element mesh is in *C* is refined (*Inset*) around the rupture tips to capture the stress singularities. (*D*) spatial distribution of ice mélange (red) deduced from the TDX digital elevation model using a threshold 250-m thickness within the active rifts 1 to 4 colored green.

Our model requires a full specification of the ice shelf geometry. The mesh domain (Fig. 2 *A*–*C*) corresponds to the geometry of year 2014 (Fig. 1 *B* and *F*) with boundaries set upstream of the grounding line position. Major active rifts (modeled as zero-width singularities) are positioned where large gradients in surface velocity across each rift are detected from interferometric synthetic-aperture radar (SAR) data (green lines in Fig. 2 *A*–*C*). Eleven active rifts are identified north of Bawden Ice Rise (BIR) along the Jason Peninsula, in the shear zone downstream of the Churchill Peninsula. Only three active rifts are identified upstream of GIR, with four additional rifts that have strong imprints in the surface elevation but no detectable imprint on the surface velocity (Fig. 1 *C*, *D*, and *F* and solid black lines in Fig. 2 *A* and *B*) and therefore are considered to be inactive. All rifts are captured within a geometrical mesh that is anisotropically adapted to best fit shear stresses at the surface. The stresses are determined from measurements of surface velocity (Fig. 1*F*) between 1 July 2014 and 31 June 2015 using Sentinel-1a interferometry SAR data (62). Each rift generates a stress singularity at both rupture tips, which requires improving the spatial resolution to within a radius of 1 to 2 km around the rupture tips where we apply a finer spatial resolution of 5 m (Fig. 2*C*). The overall anisotropic mesh has 51,311 elements, with a resolution ranging from 5 m at the rupture tips to 4 km in broad areas with no shear.

The ice shelf thickness is constrained by a high-quality TandemX (TDX) digital elevation model (DEM) (76) from 2013 and 2014, referenced to mean sea level, converted to ice shelf thickness assuming hydrostatic equilibrium, and constrained by existing ice thickness measurements from radar sounding and a modeling of the firn correction (77). Given the relative stability of the ice shelf thickness over the time period 2014 to 2017 (78), this DEM is representative of the ice shelf configuration at the time of the surface velocity (2014 to 2015). Temperature in the ice is held constant throughout the thickness (SSA formulation has no vertical gradient) and taken as 4 °C less than the annual surface temperature from the regional climate model Regional Atmospheric Climate MOdel2 (RACMO2) (79). As ice rheology is critical in constraining the flow of Larsen C (45, 80, 81), we invert for the ice rigidity, B, using the observed surface velocity as a constraint. The inversion captures the presence of active rifts (Fig. 2*B*) and yields a smoother rigidity pattern than prior inversions that ignored the presence of rifts (81). This improvement is due to the modeling of the impact of rifts in absorbing velocity jumps across flanks (69). The overall misfit between model and observations is better than 50 m/y (Fig. 2*A*).

Several forward runs are carried out where we vary the ice shelf thickness and the thickness of the mélange. Here, we define ice mélange as any part of the ice shelf where ice thickness is less than 250 m, which results in a regional map of accreted ice similar to what ref. 24 refers to as “marine-ice bodies.” Fig. 2*D* shows the extent of this area, mainly north of BIR and upstream of GIR. Due to the high resolution of our mesh, we even capture the presence of icebergs trapped in the mélange, which probably originate from the rift formation decades ago. Three sets of SSA runs are carried out: 1) Only the ice shelf (excluding the mélange) thickness is reduced, 2) only the mélange thickness is reduced, and 3) both the ice shelf and ice mélange thickness are reduced. For each run, we reduce the corresponding thickness (both ice shelf and mélange) by 1-m increments while maintaining hydrostatic equilibrium. A threshold of 1 m thickness is enforced to ensure that the model does not run into numerical singularities. A typical rate of growth of sea ice over the winter season is about 1 m (82). For each run, we calculate the stress balance of the ice shelf, the horizontal velocity field everywhere in the domain, and in particular the resulting opening rates of the rift that generated A68. This rift comprises two smaller rifts close to GIR. The eastward rift (no. 2 in Fig. 2*B*) is the rift that eventually propagated laterally along the ice front to give birth to A68. The rift is modeled as a fault line whereas in reality the rifted region is 2 to 3 km wide. As a result, the model does not perfectly match the observations along rift 2.

## Results

**Fig. 3.**
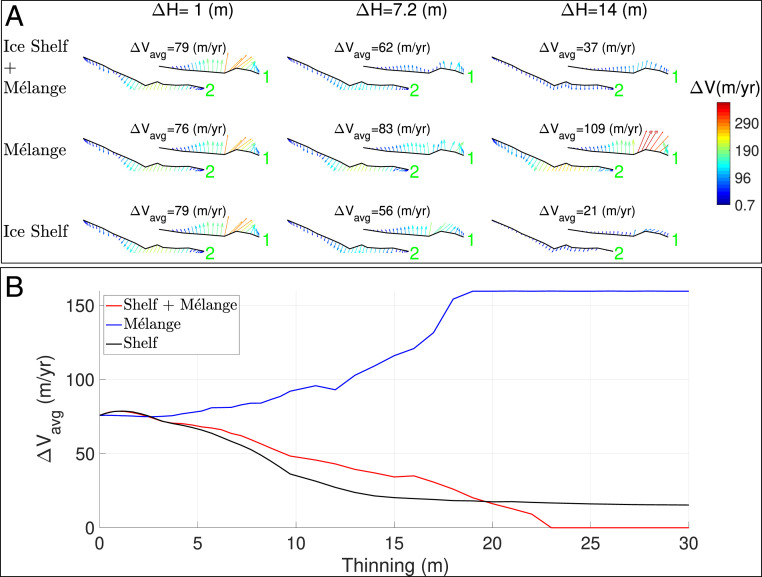
(*A* and *B*) Modeling of the combined opening rate, ΔV (m/y), of rifts 1 and 2 (see Fig. 2 for the numbering of rifts) on Larsen C Ice Shelf, Antarctica with three scenarios: 1) thinning of the ice shelf and ice mélange (ice shelf + mélange), 2) thinning of the mélange only (mélange), and 3) thinning of the ice shelf only (ice shelf) and three values of forced thinning: ΔH = 1, 7.2, and 14 m. The magnitude of the flank-to-flank opening rate is color coded from 0.48 to 350 m/y from blue to red in *A*, with a direction of opening indicated by an arrow. The opening rate of rifts 1 and 2 as a function of thinning from 0 to 30 m is shown in *B* for the three scenarios.

**Fig. 4.**
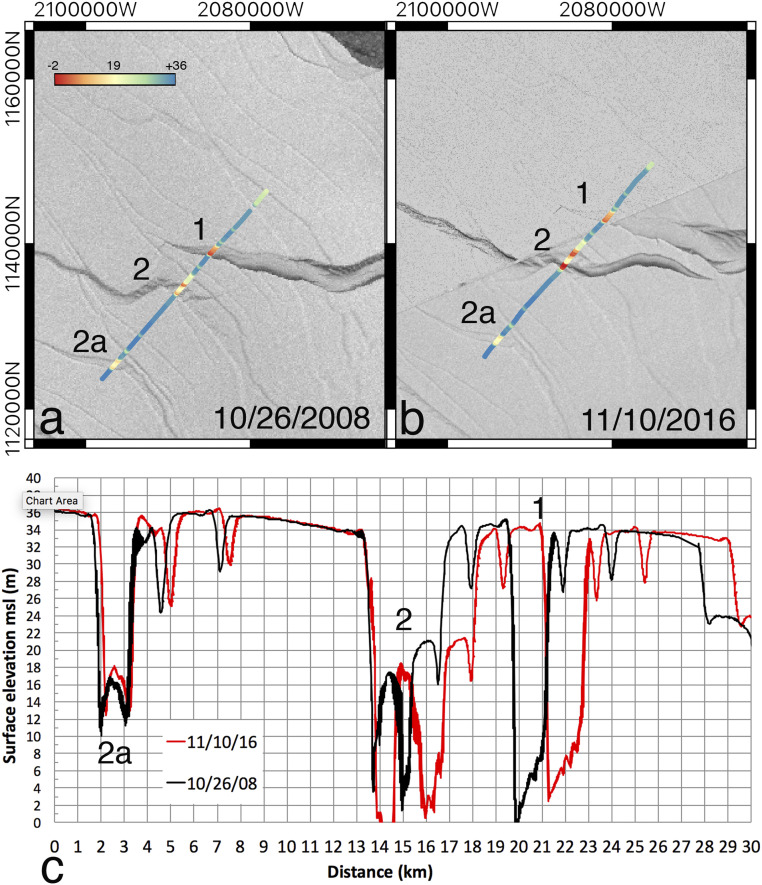
(*A* and *B*) Surface elevation (in meters above sea level) from Operation IceBridge laser altimetry in (*A*) 26 October 2008 versus (*B*) 10 November 2016 across the rifted zone of Larsen C, with annotated rifts 1, 2, and 2a from Fig. 1. (*C*) Comparison of the elevation profiles after adjustment for the 8-y motion of the ice at starting point near rift 2a versus distance (toward the north) in kilometers. Background radar imagery is from Envisat ASAR in 2008 and Sentinel-1a SAR in 2016.

A decrease in ice shelf thickness and mélange thickness leads to a decrease in the average opening rate for the GIR rift from 79 m/y (with 1 m thinning) to 36 m/y (with 14 m thinning) (Fig. 3). This decrease is homogeneous along the entire rift, with opening vectors exhibiting a small range of variation both in orientation (mainly perpendicular to the rift) and in magnitude (Fig. 4*A*). The opening rate tends to zero (Fig. 4*B*) if the ice shelf keeps thinning, hence revealing a progressive deactivation of the rift as the ice shelf and mélange thin.

A similar set of results is obtained for the case where we thin only the ice shelf (i.e., the ice mélange is unchanged), with an average opening rate decreasing from 79 to 22 m/y. Hence, the main cause of the deactivation of the rift in the prior simulation is ice shelf thinning.

Finally, we examine the scenario where only the ice mélange is thinned. We find that the opening rate increases from 76 to 112 m/y, with strong variations of the rate along the rift. Opening rates for the eastward rift are largest in the middle of the rift at 250 m/y. Opening rates for the rift closest to GIR reach upward values of 400 m/y at the tip near the ice rise. We find that the opening rate of the eastern rift saturates at 150 m/y for an overall thinning greater than 18 m. Further thinning does not increase the opening rate, which remains at 150 m/y. Thinning of the ice mélange therefore exerts a direct control on rift propagation and increases the rate of opening as the mélange thins. Beyond a threshold thickness of the ice mélange, the system reacts as if the mélange no longer existed.

Laser altimetry data collected by NASA Operation IceBridge in 2008 and 2016 along a flow line that crosses the rifted zone provide information on the evolution of the ice mélange in the intervening 8 y after compensation for the horizontal motion of the ice shelf (Fig. 4). Rift 2a is filled with a mélange that stands 12 m above sea level versus 36 m on the surrounding ice shelf, but the mélange was only 2 to 4 m above sea level in 2008 in rift 2 (20 to 40 m thick if we neglect the firn layer) and 0 to 6 m in rift 1 (0 to 60 m thick). By 2016, the mélange in 2a thickened by 10 m (1 m change in elevation) and did not change in width. Conversely, a new 1-km-wide crack with no mélange appeared in the southern side of rift 2, and the next crack to the north thinned by 20 m (2 m change in elevation) and widened by 1 km. Similar to rift 2a, rift 1 accumulated 20 m of ice in 8 y (2 m change in elevation) and maintained the same width. These observations are consistent with thinning of the mélange in rift 2 and widening of rift 2 as the ice block corresponding to iceberg A68 started to rotate off GIR.

## Discussion

Our results demonstrate a strong sensitivity of the opening of the A68 rift to changes in ice mélange thickness close to GIR. Conversely, they suggest a lesser and even negative impact of ice shelf thinning on the opening rate. Ice shelf or ice mélange thicknesses are strong contributors to the buttressing capability of an ice shelf (3, 48) based on their ability to transfer stresses through the material thickness, so we expected a priori that all three scenarios would lead to a destabilization of the rifts. The model, however, indicates that ice mélange is the main control of the stability of the A68 rift and, by extension, of the Larsen C Ice Shelf itself. This result is consistent with prior studies (34, 35, 44), where the role of the ice mélange in stabilizing Hemmen Ice Rise was identified prior to the calving of A38 in October 1998. At the time, no quality DEM, thickness (including mélange), and velocity data were available to formulate a precise diagnosis, so the study mostly addressed the two end members of the ice shelf configuration: one with the rifts filled with thick ice and one with rifts filled with open water. With ice mélange encased between the rifts, especially between the ice rise and the active rupture tip, we found that stresses are transmitted between the side margin of the embayment and the shelf and enable a solid rotation of ice blocks around the ice rise, which in turn rifts the ice shelf. In the presence of open water, the stresses are no longer transmitted, the block rotation ceases, and the rift does not propagate (69).

We have a similar situation here with the calving of A68 around GIR. But our approach quantifies this physical process in more detail because we evaluate the impact of the rate of ice mélange thinning instead of examining the end states “thick mélange” versus “no mélange.” Our simulation explains why the rift that originated in the GIR area may have become unstable following a prolonged period of mélange thinning, either from above (warmer air temperature) or from below (warmer ocean temperature), or both, or from mechanical failure of the mélange (Fig. 4), which resulted in the reactivation of the rift. In our simulations, we find that below a reduction of 18 m in ice mélange thickness, it does not matter whether the rift is filled with ice mélange or open water: The rift undergoes significant opening (at a rate of 150 m/y) and the rupture tip propagates. This evolution of the rift does not require the presence of melt water at the ice shelf surface or in the ice mélange.

The hydrofracture mechanism induced by water ponding at the surface (10–12) requires vigorous surface melt. Hydrofracture will fracture thinner ice shelves more easily than thicker ice shelves because the surface cracks will need to propagate vertically on a shorter distance to reach sea level. Our mechanism does not require water ponding, may precede extensive water ponding, and operates more effectively on thicker ice shelves. In fact, in our study, we find that ice shelf thinning is conducive to the closing of the rifts. In that case, the stresses are less well transmitted along the shear margins, block rotation may cease around GIR, and the rift is no longer propagating. A similar situation is expected on other ice shelves along ice front margins located along the diverging sides of an embayment, which is usually where icebergs detach and ice fronts stabilize.

The ice mélange is typically one order of magnitude thinner than the ice shelf proper (Fig. 4) and hence barely able to transmit stresses across rift flanks. Our model suggests a nonlinear behavior of the mechanical stability of the ice shelf as the ice mélange thins. Below a threshold value of 18 m thinning, the ice mélange ceases to transmit stresses and the rift propagates as if it were filled with open water. A sufficient condition for destabilizing an ice shelf is therefore a mechanical breakup of the ice mélange or its melting from atmospheric and oceanic processes, after which it is no longer effective at protecting the ice shelf. Our results align with past works that attribute ice shelf destabilization (following rift propagation) to the loss of accreted ice in rifts and shear zones surrounding the GIR area (24, 29). Our conclusions are also compatible with scenarios of wave energy transmission described in ref. 60 as the ice mélange may thin if sea ice formation is reduced for a number of years and the ice front will be exposed to enhanced wave-induced stress.

Strong oceanic circulation in the GIR area may have melted accreted marine ice (32, 83). This possibility was discussed in ref. 31, figure 3a, where any regime change toward the incursion of warmer modified Weddell deep water (MWDW) into the Larsen C cavity was seen to curtail basal ice accretion and its stabilizing influence. The authors showed that MWDW could potentially impact Larsen C suture zones and destabilize the ice shelf (see ref. 31, figure 2b).

At present, we do not have sufficient information about the time evolution of the ice mélange within the rifts, especially over time scales of decades, and about the surface and ocean heat fluxes that control the growth of the mélange to identify which physical processes may have reduced its thickness. We recommend more studies of the ice mélange in the future to better understand its time evolution and its impact on ice shelf stability.

We posit that the physical processes that control the stability of nascent rifts in the Peninsula are the same that operate on ice shelves farther south. An important aspect of the ice mélange is that it could start thinning independent of melt water ponding at the surface of ice shelves, for instance as the annual sea ice cover starts receding (60), possibly decades before hydrofracture. If correct, this process would explain why the ice front of Prince Gustav Channel started to retreat decades before its collapse, Larsen A started to retreat 25 y before its collapse, and Larsen B started to retreat about 16 y prior to its collapse attributed to hydrofracture, at a time when surface melt and water ponding were not as extensive in time and space (10, 16, 17, 22), but regional climate warming could have already thinned the ice mélange in and around preexisting rifts.

## Conclusions

We present a modeling study of Larsen C conducive to the calving of iceberg A68 that provides insights into the physical processes responsible for the calving. We find a strong relationship between the thickness of ice mélange near GIR and the opening rate of the rift responsible for the calving of iceberg A68. Conversely, we find that ice shelf thinning cannot explain the propagation of the rift. In fact, ice shelf thinning has the opposite effect of stabilizing the rifts. Given that ice mélange thickness depends on the ocean circulation underneath ice shelves and on radiation fluxes at the ice surface, our analysis may offer a link between climate forcing and ice shelf stability that has not been brought up to prominence and does not require extensive melt water ponding at the ice shelf surface. We posit that the ice mélange in nascent rifts near ice shelf fronts of the Antarctic Peninsula may thin decades prior to hydrofracture, thereby explaining why ice shelf fronts started to retreat well before the point of collapse through hydrofracture or reaching the compressive arch. Numerical models currently assume that ice shelves are at risk only if melt water ponding occurs. We find that a sufficient condition for their retreat could be the thinning of the ice mélange. Further investigation of the ice mélange is warranted to elucidate its role and evolution in more detail.

## Data Availability

Digital files of bed topography, rift boundaries, ice velocity, finite-element mesh, model setup, and inverted rheology data have been deposited in Dryad UC Irvine (https://doi.org/10.7280/D1TX1F).
